# Negative Poisson's ratio in 1T-type crystalline two-dimensional transition metal dichalcogenides

**DOI:** 10.1038/ncomms15224

**Published:** 2017-05-25

**Authors:** Liping Yu, Qimin Yan, Adrienn Ruzsinszky

**Affiliations:** 1 Department of Physics, Temple University, Philadelphia, Pennsylvania 19122, USA

## Abstract

Materials with a negative Poisson's ratio, also known as auxetic materials, exhibit unusual and counterintuitive mechanical behaviour—becoming fatter in cross-section when stretched. Such behaviour is mostly attributed to some special re-entrant or hinged geometric structures regardless of the chemical composition and electronic structure of a material. Here, using first-principles calculations, we report a class of auxetic single-layer two-dimensional materials, namely, the 1T-type monolayer crystals of groups 6–7 transition-metal dichalcogenides, MX_2_ (M=Mo, W, Tc, Re; X=S, Se, Te). These materials have a crystal structure distinct from all other known auxetic materials. They exhibit an intrinsic in-plane negative Poisson's ratio, which is dominated by electronic effects. We attribute the occurrence of such auxetic behaviour to the strong coupling between the chalcogen *p* orbitals and the intermetal *t*_2g_-bonding orbitals within the basic triangular pyramid structure unit. The unusual auxetic behaviour in combination with other remarkable properties of monolayer two-dimensional materials could lead to novel multi-functionalities.

## Abstract (web summary)

Auxetic materials—those that expand laterally when stretched—can possess enhanced mechanical properties, including shear modulus, indentation resistance, and fracture toughness. Here the authors show that 1T-type crystalline 2D transition metal dichalcogenides exhibit intrinsic in-plane negative Poisson's ratios.

The Poisson's ratio of a material characterizes its response to uniaxial load and is given by *ν*_*ab*_*=−ɛ*_*b*_/*ɛ*_*a*_, where *ɛ*_*a*_ is an applied strain in the *a* axis direction and *ɛ*_*b*_ is the resulting strain in a transverse *b* axis direction. Counter-intuitively, negative Poisson's ratio (auxetic) materials^1^ expand laterally when stretched and contract laterally when compressed. They can lead to enhanced mechanical properties, such as shear modulus^2^, indentation resistance^3^ and fracture toughness^4^. The unusual auxetic effect itself and concomitant enhancements in other material properties offers enormous potential in many technologically important applications^5^,^6^,^7^, such as biomedicine^8^, sensors^9^, fasteners^10^ and protective equipments^11^.

Auxetic effect has been reported in a number of natural and man-made materials and structures in bulk form^5^,^6^,^12^,^13^, for example, cubic metals^14^,^15^, α-cristobalite (SiO_2_)^16^, α-TeO_2_ (ref. 17), the zeolite mineral natrolite^18^, honeycombs^19^, foams^7^, microporous polymers^20^,^21^, composites^22^,^23^, ceramics^24^, molecular auxtics^25^, metal-organic frameworks^26^, bucklicrystals^27^ and origami structures^28^,^29^,^30^. Geometric considerations dominate the literature in understanding such auxetic effects and designing new auxetic materials. For most of these auxetic materials, the auxetic effect is explained by some special re-entrant structure or the crystal structure that can be viewed as being made up of rigid building blocks linked by flexible hinges^1^,^19^,^31^,^32^,^33^, independent of their chemical composition and electronic structure.

Auxetic effect has also been recently reported in several monolayer two-dimensional (2D) materials. For example, the out-of-plane negative Poisson's ratio was discovered in phosphorene^34^,^35^, GeS^36^ and monolayer arsenic^37^. The in-plane negative Poisson's ratio was also predicted in borophene^38^ and three theoretically proposed but not-yet-synthesized materials (that is, the penta-graphene^39^,^40^, *hα*-silica^41^ and Be_5_C_2_ (ref. 42)). Similar to that in the bulk auxetic materials, the auxetic behaviour in these 2D materials is also considered to originate mainly from the puckered or buckled crystal structure.

In this study, using quantum mechanical first-principles calculations (see Methods section), we report a class of auxetic single-layer 2D materials with an intrinsic in-plane negative Poisson's ratio. They differ from other known auxetic materials not only in their crystal structure but also in the microscopic origin of auxetic behaviour. These materials are the 1T-type crystalline monolayers of groups 6–7 transition metal dichalcogenides, 1T-MX_2_ (M=Mo, W, Tc, Re; X=S, Se, Te). In contrast to those known bulk or 2D auxetic materials, the in-plane auxetic behaviour discovered in groups 6–7 1T-MX_2_ cannot be explained merely from their geometric structure because the non-auxetic behaviour is also found in other groups of MX_2_ compounds with the same 1T-type structure. This dichotomy between auxetic and non-auxetic behaviour in the 1T-MX_2_ compounds is explained by their distinct electron structures. The in-plane stiffness of those 1T-MX_2_ materials is predicted to be order of 10^2^ GPa, at least three orders of magnitude higher than man-made auxetic materials. The high in-plane stiffness and the auxetic behaviour in combination with other remarkable electronic and optoelectronic properties of the single-layer 2D materials^43^ could lead to novel multi-functionalities, such as nanoscale auxetic electrodes and sensors.

## Results

### Crystal structure

**Figure 1:**
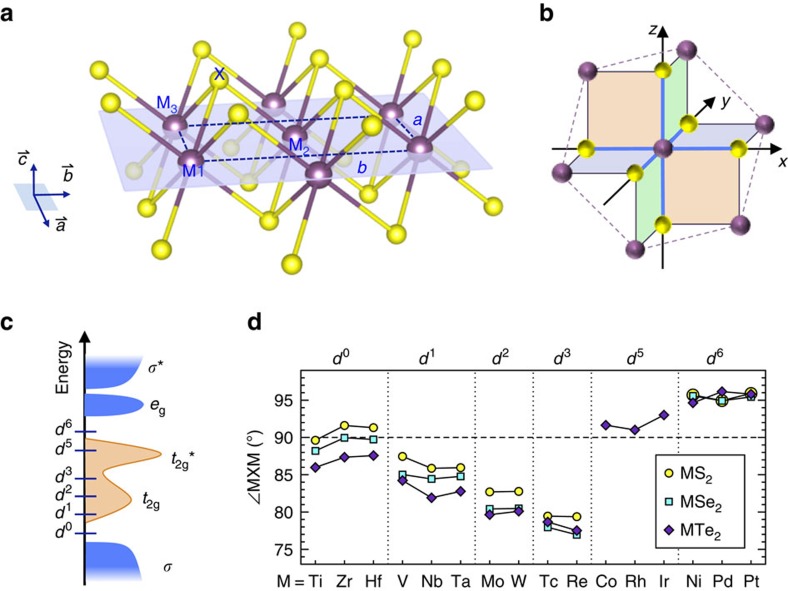
Structure of monolayer 1T-MX_2_. (**a**) Crystal structure. The basic X-M_1_-M_2_-M_3_ triangular pyramid unit is marked. The rectangular outline displays the unit cell adopted in our calculation. It contains two MX_2_ formula units. (**b**) Local structure of M-centred octahedron. The metal atoms form three one-dimensional chains in the directions of *y=x*, *z=y* and *z=*−*x* in the local reference frame. The M–M interaction is through the *t*_2g_-orbital coupling. (**c**) Schematic configuration of DOS showing the gradual filling of *d* orbitals from group 4 (*d*^0^) to group 10 (*d*^6^) 1T-MX_2_. The horizontal bars denote the corresponding Femi level of the system. *t*_2g_ and *t*_2g_* correspond to the intermetal *t*_2g_-bonding and *t*_2g_-antibonding states, respectively. (**d**) Predicted M–X–M bond angles in the relaxed structure of strain-free 1T MX_2_. Note, in the triangular pyramid as shown in Fig. 1a, ∠M_1_XM_2_=∠M_2_XM_3_=∠M_3_XM_1_=∠MXM.

The single layers of 2D transition metal dichalcogenides are formed by a hexagonally packed layer of metal (M) atoms sandwiched between two layers of chalcogen (X) atoms (Fig. 1). Each chalcogen atom forms the apex of a triangular pyramid that has three metal atoms at its base. The symmetry of the chalcogen array about each metal atom is either octahedral or trigonal prismatic. The former is often referred to as the 1T phase, whereas the latter as the 1H phase. Depending on the combination of the metal and chalcogen elements, one of the two phases is thermodynamically preferred. Most group-6 MX_2_ compounds thermodynamically prefer the 1H phase^44^, but the metastable 1T phase is also observed^45^,^46^,^47^. For other groups of layered MX_2_ compounds, most crystallize in the high-symmetry 1T or low-symmetry distorted 1T phase^44^,^48^. The 1H-MX_2_ compounds are known to be non-auxetic in the plane due to their hexagonal in-plane crystalline structure. We hence focus on 42 monolayer MX_2_ compounds in the high-symmetry 1T phase (Table 1).

### Poisson's ratio results

**Figure 2:**
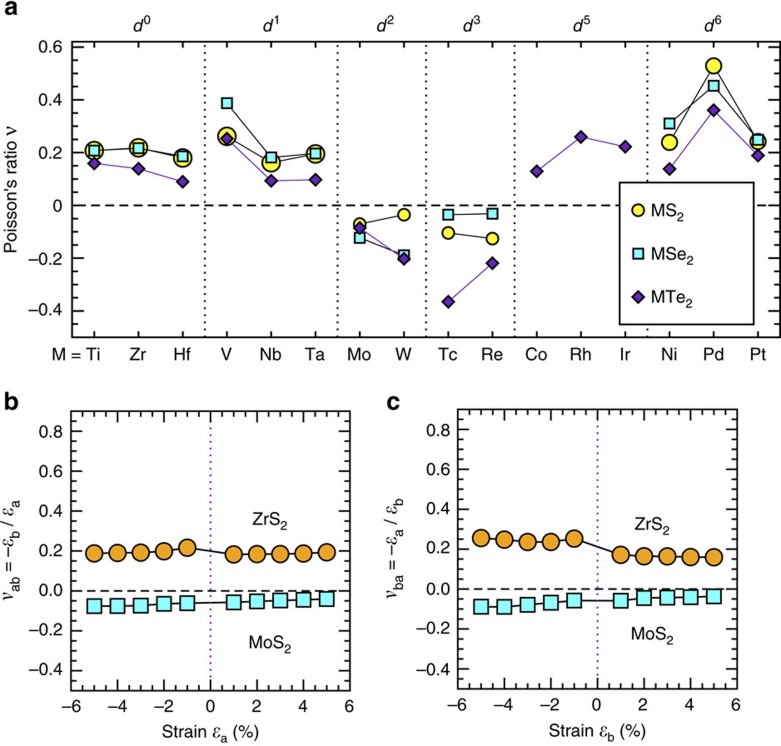
Poisson's ratios. (**a**) Poisson's ratio, *ν*_*ab*_=−*ɛ*_*b*_/*ɛ*_*a*_, calculated for a 5% strain applied along the *a* axis (that is, *ɛ*_*a*_=5%). (**b**,**c**) Poisson's ratios for ZrS_2_ and MoS_2_ as a function of strain applied along the *a* axis (**b**) and *b* axis (**c**).

Figure 2a shows our calculated Poisson's ratio results (*ν*_*ab*_) for 42 1T-MX_2_ compounds in the *b* axis direction subjected to a 5% tensile strain applied along the *a* axis direction. Remarkably, we find that the sign of Poisson's ratio strongly depends on the *d*-electron count. All 12 1T-MX_2_ compounds from group 6 (*d*^2^) and group 7 (*d*^3^) exhibit negative Poisson's ratios, ranging from −0.03 to −0.37. Seven of them (that is, TcTe_2_, ReTe_2_, WTe_2_, WSe_2_, MoSe_2_, ReS_2_ and TcS_2_) have a Poisson's ratio <−0.1, higher in magnitude than that of borophene (−0.04 along *a* and −0.02 along *b*)^38^, rendering them more promising candidates for specific applications in mechanical nanodevices. For other groups of 1T-MX_2_ compounds, we find positive Poisson's ratios ranging from 0.09 to 0.53.

Figure 2b,c shows our calculated Poisson's ratios (*ν*_*ab*_ and *ν*_*ba*_) as a function of applied strain in two example compounds, non-auxetic ZrS_2_ and auxetic MoS_2_. For both compounds, Poisson's ratio varies slowly as applied strain goes from −5% to 5%, suggesting a dominant linear elastic behaviour within the strain range considered. (Note the Poisson's ratio at a large strain (that is, >5% or <−5%) may strongly depend on the strain. This behaviour is not pursued in this work since such large strains are often experimentally inaccessible.) The small differences between *ν*_*ab*_ and *ν*_*ba*_ reflect a nearly isotropic auxetic or non-auxetic behaviour inside the 1T-structure plane. Therefore, the *d*-electron count dependence of the sign of Poisson's ratio as shown in Fig. 2a does not change with respect to the amount of the applied strain within the linear elastic range and the loading direction inside the plane.

### Stiffness

To compare the stiffness (Young's modulus) of a single-layer material with bulk materials, we calculate its 3D in-plane stiffness (*Y*_3D_) from 2D in-plane stiffness (*Y*_2D_) and effective layer thickness (*t*) via *Y*_3D*=*_*Y*_2D_/*t*. The *Y*_2D_ is directly derived from first-principles total energies as a function of uniaxial strain. The effective layer thickness *t* can also be uniquely determined from first-principles calculated bending energy^49^. Here for simplicity, we approximate *t* as *t=t*_0_+0.8 Å, where *t*_0_ is the distance between the top and bottom chalcogen atom layers and the 0.8 Å is the total effective decay length (0.4 Å in each layer side) of electron density into the vacuum. The 0.8 Å is derived from the first-principles calculated layer thickness for 1H-MoS_2_ (ref. 49). MSe_2_ and MTe_2_ may have different decay lengths than MS_2_. However, such difference should be less than one time of magnitude. Hence, using a different decay length does not induce one time of magnitude difference in the calculated 3D in-plane stiffness.

Table 1 shows that the 3D in-plane stiffness of almost all 1T-MX_2_ compounds lies in between 100 and 300 GPa. Among the auxetic *d*^2^–*d*^3^ 1T-MX_2_ compounds, WS_2_ and ReSe_2_ are the stiffest, having a stiffness of ∼290 GPa; TcTe_2_ is the softest, having a stiffness of ∼80 GPa. Man-made auxetic materials typically have a stiffness in the range from ∼10^−5^ to ∼1 GPa, and naturally occurring auxetic bulk solids exhibit a stiffness of ∼10^1^–10^2^ Pa (ref. 50). Therefore, even considering the uncertainty of our calculated 3D stiffness (less than one order of magnitude) that may be caused by using different approximations for effective layer thickness, the 3D stiffness values predicted for 1T-MX_2_ compounds are among the highest in the naturally occurring crystalline solids and are at least three orders of magnitude higher than man-made auxetic materials.

The fact that both auxetic and non-auxetic materials are found in the same 1T-structure type implies that the auxetic effect is not a purely geometric property. The *d*-electron count dependence of electronic structure must be involved. In the 1T structure, the *d* orbitals of the octahedrally coordinated transition metal split into two groups, *d*_*xy*,*yz*,*zx*_ (*t*_2g_) and 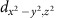 (*e*_g_). In what follows, we shall show that (i) transition metals interact with each other through *t*_2g_-orbital coupling, and (ii) the coupled *t*_2g_ orbitals are further coupled with the lone-pair electrons of chalcogen atoms. It is the gradual filling of such *t*_2g_*-p*-hybridized bands that leads to the different behaviour of Poisson's ratio.

### Intermetal *t*_2g_-orbital coupling

In the ideal 1T phase, the M-centred octahedra share edges, forming three one-dimensional M-chains along the directions of lines *y=x*, *y=z*, and *z=*−*x*, respectively, within the local reference frame of the octahedra (Fig. 1b). The metal atoms can interact with each other through the coupling between their *t*_2g_ orbitals. This coupling gives rise to *t*_2g_-bonding states and *t*_2g_-antibonding states, with no energy gap in between due to the weak coupling nature. The *t*_2g_ states are mostly located within the gap between the bonding and antibonding bands of the M–X bonds (Fig. 1c).

The progressive filling of these *t*_2g_ bands from group 4 (*d*^0^) to group 10 (*d*^6^) species leads to different M–M bonding or antibonding character at the Fermi level. In *d*^1^-*d*^3^ 1T-MX_2_, the Fermi level crosses the *t*_2g_-bonding states; the highest occupied bands close to the Fermi-level thus exhibit a stronger bonding character as we go from *d*^1^ to *d*^3^. This bonding character attracts the metal atoms towards each other, leading to an intermetal distance shorter than that in the ideal 1T structure. In *d*^*5*^–*d*^*6*^ 1T-MX_2_, since the *t*_2g_ bonding states can accommodate up to six electrons (three from each metal), all *t*_2g_-bonding states are filled and the Fermi level crosses the *t*_2g_-antibonding states. Hence the highest occupied bands in the vicinity of the Fermi level exhibit antibonding character, repelling metal atoms from each other.

The existence of the intermetal *t*_2g_-orbital interactions is reflected by the *d*-electron count dependence of the M–X–M bond angles (∠MXM) as illustrated in Fig. 1d. The ideal 1T phase has regular octahedra with ∠MXM=90°. In the *d*^0^ 1T-MX_2_ compounds, the ∠MXM deviates least from 90°. This is expected since all *t*_2g_ states are almost completely unoccupied and the intermetal *d*–*d* interaction is marginal. For the *d*^1^–*d*^3^ 1T-MX_2_, all have acute ∠MXM, decreasing with the increasing *d*-electron count. This trend arises from the increasing intermetal *t*_2g_-bonding character in going from *d*^1^ to *d*^3^, which shortens the intermetal distance. In the *d*^5^–*d*^6^ 1T-MX_2_, the ∠MXM jumps up to over 90°, consistent with the intermetal *t*_2g_-antibonding character.

Figure 1d also shows that the chalcogen atoms have minor effect on ∠MXM compared with the transition metals with different *d*-electron counts, but a trend can still be observed: the ∠MXM decreases with increasing atomic number of the chalcogen. For example, the ∠MXM of TiS_2_, TiSe_2_ and TiTe_2_ decreases from 89.6° to 88.2° to 86.0°. This trend is not associated with the intermetal *t*_2g_-orbital interaction; instead it is intrinsic to the spatial distribution of the lone-pair charge density relative to that of the M–X bonds around the chalcogen.

### *t*_2g_–*p*-orbital coupling

**Figure 3:**
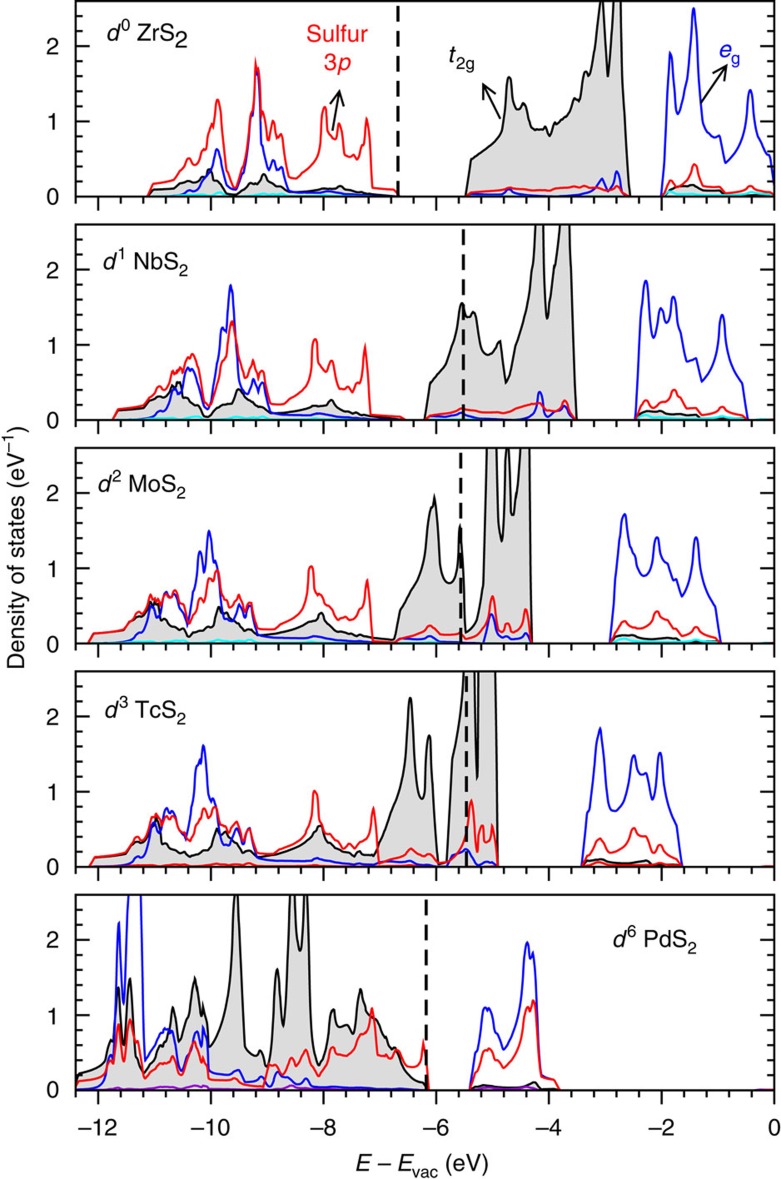
DOS of 4*d* MS_2_ in the ideal 1T structure. The *t*_2g_*-p* orbital coupling manifests itself in the overlap of their DOS. The local reference frame in the octahedral is used for projecting DOS. The DOS shown in the figure are *t*_2g_=*d*_*xy*_+*d*_*yz*_+*d*_*zx*_, *e*_g_=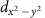
+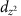 and *p=p*_*x*_+*p*_*y*_+*p*_*z*_. The vertical dashed lines show the position of Fermi level. The energy is aligned to the vacuum level.

The intermetal *t*_2g_ orbitals are further coupled with chalcogen *p* orbitals in 1T-MX_2_. It can be seen from their projected density of states (DOS) as shown in Fig. 3. In the 4*d* transition metal disulfides with the ideal 1T structure, we find that the DOS of sulfur 3*p* and metal *t*_2g_ states overlap, as manifested by their similar DOS peak shapes and positions in energy. The *t*_2g_–*p-*orbital overlap is marginal in *d*^0^ ZrS_2_, but it increases quickly in going from *d*^1^ NbS_2_ to *d*^6^ PdS_2_. This trend is clear not only in the energy range from −12 to −7 eV, where the major peaks of 3*p* DOS are located, but also near the Fermi level.

The *t*_2g_–*p*-orbital interaction is attractive because the X ligand has one lone electron pair and acts as a sigma donor. In *d*^1^–*d*^3^ MX_2_, the *t*_2g_–*p* coupling force draws atom X towards the intermetal bond centres, because the *t*_2g_ states are the intermetal bonding states spreading over the M–M bond centers. In *d*^5^–*d*^6^ MX_2_, the *t*_2g_–*p* coupling force attracts atoms M and X towards each other, because the *t*_2g_ states are antibonding and localized near the metal atoms. The *d*-electron count dependence of *t*_2g_–*p* interaction direction plays a key role in determining the structure deformation presented below.

### Deformation mechanism

**Figure 4:**
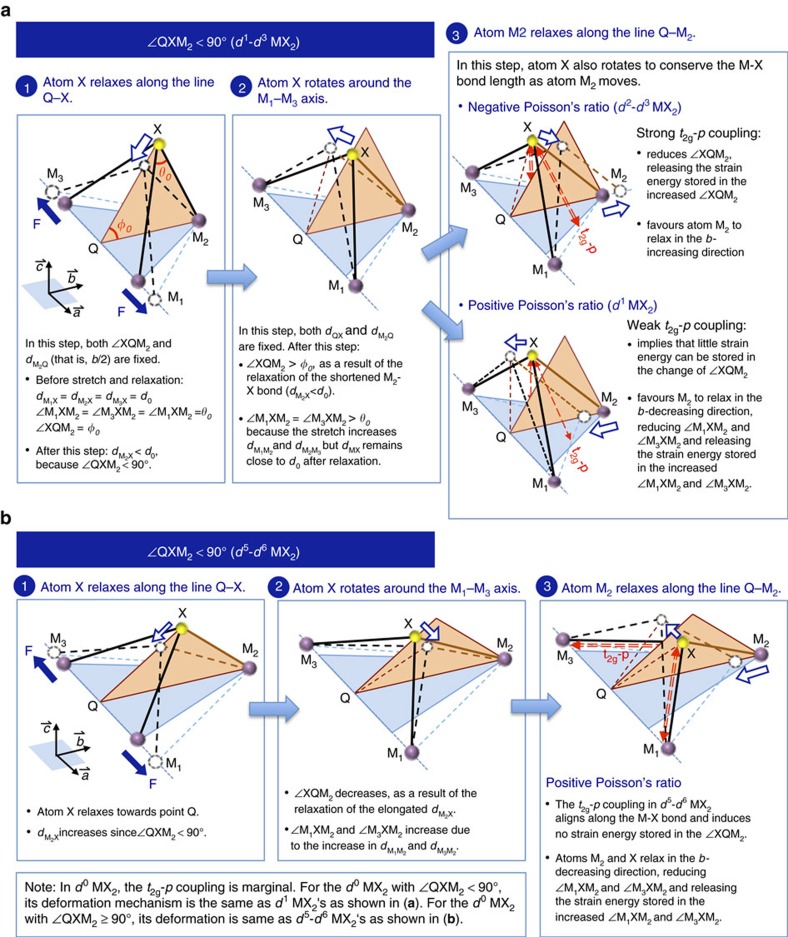
Deformation mechanism. The solid and dashed M–X bonds indicate, respectively, the initial and final configurations at each relaxation step. The force is applied along the lattice-*a* direction. The red dashed arrows indicate the direction of the *t*_2g_*-p* orbital interaction. The hollow blue arrows show the resulting movement of the X and M_2_ atoms within the Q–X–M_2_ plane.

To understand the microscopic origin of Poisson's ratios, let us now look into the resulting structural relaxation subjected to a tensile strain applied along the *a* axis. Due to the centrosymmetric nature of the 1T phase, the whole relaxation process manifests itself in the triangular pyramid unit as illustrated in Fig. 4. For the stretch along the M_1_–M_3_ axis (that is, axis *a*), the resulting relaxation involves only atoms M_2_ and X moving inside the Q–X–M_2_ plane. Hence, two relations always hold during relaxation: *d*_M_1_M_2__=*d*_M_2_M_3__ and ∠M_1_XM_2_=∠M_3_XM_2_.

We analyse the relaxation process by decomposing it into three consecutive steps: (i) atom X relaxes along the line Q–X, (ii) atom X rotates around the M_1_–M_3_ axis, and (iii) atom M_2_ relaxes along the line Q–M_2_. In the first two steps, the lattice constant *b* is fixed to the value found in the relaxed strain-free 1T structure. In the third step, *b* varies as atom M_2_ moves along the Q–M_2_ line, leading to different Poisson's ratio behaviour.

Figure 4 shows the detailed structural relaxation in the three consecutive steps described above for 1T-MX_2_ with ∠QXM_2_<90° (Fig. 4a) and with ∠QXM_2_>90° (Fig. 4b) separately. Each step can be understood in the way that atom X (or atoms X and M_2_) relaxes to conserve the M–X bond length (*d*_MX_) since *d*_MX_ is energetically dominant. After the first two steps of the relaxation, it can be seen that (i) both *d*_M_1_M_2__ (also *d*_M_2_M_3__) and ∠M_1_XM_2_ and ∠M_3_XM_2_ (Supplementary Fig. 1) increase in all 1T-MX_2_ compounds no matter whether ∠QXM_2_ is larger or smaller than 90°, and (ii) ∠XQM_2_ increases in the 1T-MX_2_ with ∠QXM_2_<90° but decreases in the 1T-MX_2_ with ∠QXM_2_ >90° (Supplementary Fig. 1). The changes in *d*_M_1_M__2___ and *d*_M__2__M_3__, ∠M_1_XM_2_ and ∠M_2_XM_3_ and ∠XQM_2_ thus store the strain energy, which will be partially released in the subsequent third step relaxation.

The third-step relaxation determines the sign of Poisson's ratio. The negative Poisson's ratio of *d*^2^–*d*^3^ MX_2_ can be attributed to the strong *t*_2g_–*p*-orbital coupling. Such strong coupling implies a large amount of strain energy stored in the decreased ∠XQM_2_ after the second step. This part of strain energy will be released in this third step through atom M_2_ relaxing along the increased *b*-lattice direction, leading to a negative Poisson's ratio. The strength of *t*_2g_–*p*-orbital coupling depends not only on the *d*-electron count of the transition metal but also on the chalcogen atom. This dependence explains why the Poisson's ratio of the compounds from same *d*^2^ or *d*^3^ group also differs from one another as shown in Fig. 2a.

For *d*^0^–*d*^1^ MX_2_, the positive Poisson's ratio results from the marginal or weak intermetal *t*_2g_ coupling and *t*_2g_–*p* coupling. Such weak couplings imply that the strain energy stored in *d*_M_1_M__2___ and *d*_M_2_M_3__ and ∠XQM_2_ is also marginal or small. The major strain energy that can be released in the third step is thus stored in the increased ∠M_1_XM_2_ and ∠M_2_XM_3_. Therefore, it is energetically favourable that atom M_2_ relaxes in the *b*-decreasing direction, reducing the increase in ∠M_1_XM_2_ and ∠M_2_XM_3_, and resulting in a positive Poisson's ratio. For *d*^5^–*d*^6^ MX_2_, the positive Poisson's ratio originates from the fact that the *t*_2g_–*p* coupling aligns along the M–X bond and does not energetically affect the change in ∠XQM_2_. In other words, the strain energy stored in the decreased ∠XQM_2_ is small. Since the *t*_2g_ antibonding is also generally weak, the relaxation of atom M_2_ is energetically favourable in the *b*-decreasing direction, giving rise to a positive Poisson's ratio. This deformation mechanism is similar to that in *d*^0^–*d*^1^ compounds.

Simply saying, the negative Poisson's ratio in *d*^2^–*d*^3^ MX_2_ results from the strong attractive coupling between the intermetal *t*_2g_-bonding states and the X *p* states, which prevents atoms X and M_2_ relaxing toward the ∠XQM_2_-increasing direction. The positive Poisson's ratio arises from lack of such strong *t*_2g_–*p* coupling in other groups of 1T-MX_2_.

## Discussion

The monolayer MX_2_ materials involve transition metals where strong correlation effects may be not well captured by the new strongly constrained and appropriately normed (SCAN) meta-GGA functional. To check the robustness of our results, we also calculated the Poisson's ratio for 12 *d*^2^–*d*^3^ MX_2_ by using the HSE06 hybrid functional^51^. The results are summarized in Supplementary Table 1. It shows that the Poisson's ratio of eight 1T-MX_2_ compounds (that is, MoSe_2_, MoTe_2_, WSe_2_, WTe_2_, TcTe_2_, ReS_2_, ReSe_2_, ReTe_2_) remains negative, whereas for other four compounds (that is, MoS_2_, WS_2_, TcS_2_, TcSe_2_) their Poisson's ratio changes the sign from negative to slightly positive, which is still very interesting and useful for applications. Although it is found that the SCAN lattice constants agree better with experiment than the HSE06 ones for most of the compounds listed in this table, it is uncertain whether SCAN predicts a more accurate Poisson's ratio than HSE06 since the semilocal SCAN functional could make larger density-driven error in the energy than HSE06 does for the system under stretching^52^. This uncertainty calls for experimental validation and further theoretical study. Nevertheless, the auxetic behaviour we find is robust in most of the *d*^2^–*d*^3^ MX_2_ compounds. The less negative Poisson's ratio predicted by HSE06 (Supplementary Table 1) further indicates that the auxetic behaviour originates from the strong *p*–*d* coupling. In general, compared with the semilocal SCAN functional, HSE06 yields more localized metal *d* and chalcogen *p* orbitals and hence the weaker hybridization between them, which leads to less negative Poisson's ratios in HSE06.

Our predicted in-plane auxetic behaviour is intrinsic in the 1T structure without any external engineering and occurs in the elastic region. This is different from the extrinsic auxetic behaviour reported in the epitaxial oxide thin-film^53^,^54^ and the engineered 2D materials, such as the wrinkled graphene^55^, graphane^56^ and borophane^57^. Recently, the negative Poisson's ratio was also reported in metal nanoplates^58^, pristine graphene^59^ and semi-fluorinated graphene^60^. The negative Poisson's ratio claimed there corresponds to the ratio calculated from *ν*_*ab*_*=−∂ɛ*_*a*_*/∂ɛ*_*b*_ under large stains, differing from that calculated from *ν*_*ab*_*=−ɛ*_*a*_*/ɛ*_*b*_ (the original definition of Poisson's ratio) as we followed here. The Poisson's ratios calculated from *ν*_*ab*_*=−ɛ*_*a*_*/ɛ*_*b*_ for pristine graphene^59^ and semi-fluorinated graphene^60^ are actually both positive under a strain <∼15%, and for metal nanoplates, it is also positive under a strain <∼4%.

Finally, it is noteworthy that the auxetic behaviour of *d*^2^–*d*^3^ MX_2_ compounds is predicted in the high-symmetry 1T phase. This phase is known to be metastable or dynamically unstable in both *d*^2^ and *d*^3^ MX_2_ compounds^44^,^61^,^62^,^63^. However, experimentally, relevant phase diagrams of monolayer materials differ from those of bulk materials. The kinetic barriers between the different phases of monolayers may arise and be affected by many external factors, such as interfaces, underlying substrate, temperature, strain and impurities. Therefore, it is not uncommon to observe the undistorted 1T phase synthesized experimentally. For instance, although no kinetic barrier is found from first-principles calculations between the unstable 1T phase and dynamically stable distorted 1T phase, the undistorted 1T monolayer structures of MoS_2_, MoSe_2_, WS_2_ and WSe_2_ are observed from the exfoliation using Li-intercalation method^45^,^64^. For MoS_2_, the coexistence of 1T and 1H domains is also observed in the same monolayer^46^,^47^. Such heterogeneous monolayers with auxetic and non-auxetic domains are particularly intriguing since they could lead to novel functionality.

## Methods

All calculations were performed using density functional theory and the plane-wave projector augmented-wave^65^ method as implemented in the VASP code^66^. The new SCAN meta-generalized gradient approximation was used^67^,^68^. SCAN is almost as computationally efficient as PBE-GGA functional, yet it often matches or exceeds the accuracy of the more computationally expensive hybrid functionals in predicting the geometries and energies of diversely bonded systems^68^. Supplementary Table 2 shows our calculated lattice constants for 1T-MX_2_ compounds. They agree very well with available experimental data^44^, especially for groups 4–7 1T-MX_2_ whose errors are within 1%. An energy cutoff of 500 eV was used. The monolayer structure is modelled in an orthorhombic supercell that contains two formula units (Fig. 1a) and a 20 Å vacuum space inserted in the out-of-plane direction. A 24 × 14 × 1 k-point grid was used to sample the Brillouin zone during structure relaxation. All atoms were fully relaxed until their atomic forces were <0.005 eV Å^−1^. The effects of spin-orbit coupling on the structural deformation are considered to be minor and hence not included in our study.

The Poisson's ratio is calculated from the engineering strain (*ɛ*), which is defined as the change in length Δ*L* per unit of the original length *L*, that is, *ɛ=*Δ*L/L*. The applied uniaxial strain is realized in our calculations by fixing the lattice parameter to a value different from its equilibrium value during structural relaxation. The resulting strain in the transverse direction is extracted from the fully relaxed structure subjected to an applied strain.

### Data availability

The authors declare that the data supporting the findings of this study are available within the paper and its Supplementary Information files.

## Additional information

**How to cite this article:** Yu, L. *et al*. Negative Poisson's ratio in 1T-type crystalline two-dimensional transition metal dichalcogenides. *Nat. Commun.* **8,** 15224 doi: 10.1038/ncomms15224 (2017).

**Publisher's note**: Springer Nature remains neutral with regard to jurisdictional claims in published maps and institutional affiliations.

## Supplementary Material

Supplementary Information Supplementary Figure, Supplementary Tables and Supplementary References

## Figures and Tables

**Table 1:** Predicted in-plane stiffness for 42 monolayer 1T-MX_2_ compounds.

**M**^**4+**^	**X**_**2**_	**3D in-plane stiffness** ***Y***_**3D**_ **(GPa)**			**2D in-plane stiffness** ***Y***_**2D**_ **(Nm**^**−1**^**)**		
		**−S**_**2**_	**−Se**_**2**_	**−Te**_**2**_	**−S**_**2**_	**−Se**_**2**_	**−Te**_**2**_
*d*^0^	Ti	236	183	108	85	70	46
	Zr	210	182	103	77	71	44
	Hf	233	200	117	85	77	50
*d*^1^	V	263	226	159	97	88	67
	Nb	225	180	127	87	73	56
	Ta	265	215	129	101	85	57
*d*^2^	Mo	261	249	205	103	104	92
	W	289	225	199	113	94	88
*d*^3^	Tc	232	244	77	94	104	34
	Re	222	286	157	90	123	71
*d*^5^	Co			164			59
	Rh	Non-layered structure		98	Non-layered structure		37
	Ir			121			45
*d*^6^	Ni	287	248	126	89	80	44
	Pd	232	193	180	77	66	63
	Pt	296	244	208	96	82	74

The 3D in-plane stiffness (Young's Modulus) (*Y*_3D_) is calculated from 2D in-plane stiffness (*Y*_2D_) divided by the effective layer thickness *t* given by *t=t*_*o*_+0.8 Å, where *t*_*o*_ is the distance between the top and bottom chalcogen atom layers.
