# Supplementary material for: Negative Poisson's ratio in 1T-type crystalline two-dimensional transition metal dichalcogenides
Source: Nat Commun. 2017 May 25;8:15224. doi: 10.1038/ncomms15224 (PMC5458503; doi:10.1038/ncomms15224)
Supplement: Supplementary Information — Supplementary Figure, Supplementary Tables and Supplementary References [file ncomms15224-s1.pdf]

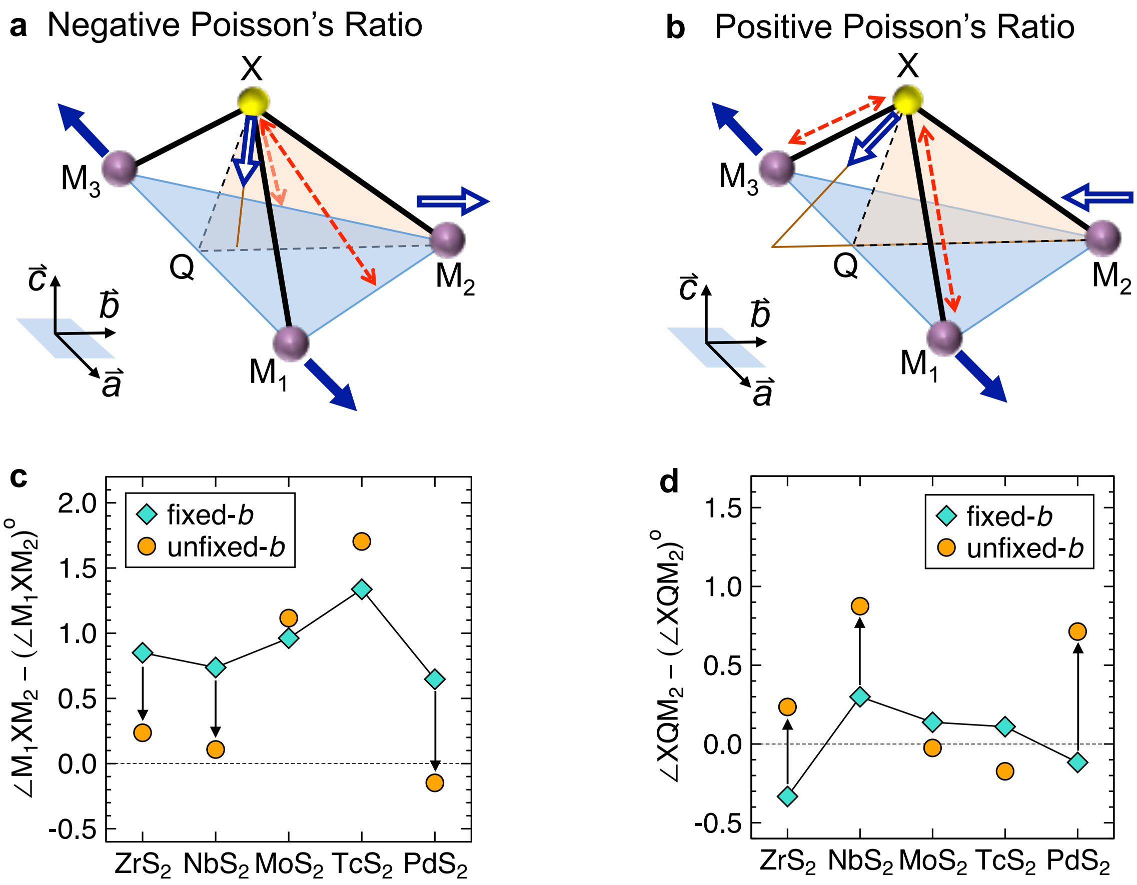


**Supplementary Figure 1 | Deformation Mechanism. a-b**, Motion of atoms in the auxetic and non-auxetic 1T-$MX_2$ compounds. The solid and open blue arrows indicate the applied force direction and resulting atom motion direction, respectively. The dashed red arrow indicates the direction of the $t_{2g}$-p orbital coupling force. **c,** Change in $\angle M_1XM_2$ after step-ii relaxation (i.e., the relaxation with the lattice *b* fixed to its value in the relaxed strain-free structure). **d**, Change in X-$M_3$-$M_1$-$M_2$ dihedral angle $\angle QXM_2$ $\angle M_1XM_2$ after step-iii relaxation. The $(\angle QXM_2)^o$ and $(\angle M_1XM_2)^o$ are the reference angles taken in the relaxed structure of corresponding strain-free $MX_2$. The diamond and circle symbols represent the angles extracted from the structures relaxed under fixed lattice-*b* and unfixed lattice-*b*, respectively, subjected to a 5% tension strain in the lattice-*a* direction. The solid lines and arrows in c-d are guides to the eyes.

**Supplementary Table 1:** The Poisson's ratios and lattice constants of groups 6-7 $MX_2$ compounds calculated from SCAN and HSE06. Available experimental lattice constants are shown in parentheses.

| | In-plane Poisson's ratio along *a*: SCAN/HSE06 | | | Lattice Constant *a* (Å): SCAN/HSE06 (Experiment) | | |
|---|---|---|---|---|---|---|
| $X_2$ \ $M^{4+}$ | -$S_2$ | -$Se_2$ | -$Te_2$ | -$S_2$ | -$Se_2$ | -$Te_2$ |
| Mo | -0.07/**0.01** | -0.12/-0.00 | -0.09/-0.01 | **3.1998/3.1657 (3.201)**[1] | **3.2685/3.2529 (3.270)**[1] | 3.4970/3.4626 (3.469)[2] |
| W | -0.04/**0.05** | -0.19/-0.05 | -0.20/-0.06 | **3.1908/3.1843 (3.222)**[1] | 3.2574/3.2645 | **3.4970/3.4759 (3.496)**[3] |
| Tc | -0.10/**0.03** | -0.04/**0.01** | -0.37/-0.02 | 3.0692/3.0255 | 3.1543/3.1386 | 3.4149/3.3486 |
| Re | -0.13/-0.02 | -0.03-0.02 | -0.22/-0.09 | 3.0750/3.0590 | 3.1311/3.1381 | 3.3834/3.3551 |

**Supplementary Table 2.** SCAN-calculated lattice constants of 42 1T-$MX_2$ compounds. Available experimental values are shown in parentheses. Note the experimental lattice constants listed for $d^5$-$d^6$ $MTe_2$ compounds are extracted from the distorted 1T structure. The small difference in lattice constant between the undistorted and distorted phases is expected.

| | | Lattice Constant *a* (Å) | | |
|---|---|---|---|---|
| $M^{4+}$ | $X_2$ | $-S_2$ | $-Se_2$ | $-Te_2$ |
| $d^0$ | Ti | 3.4055 (3.405)[4] | 3.5165 (3.535)[4] | 3.7648 (3.766)[4] |
| | Zr | 3.6833 (3.662)[4] | 3.7815 (3.770)[4] | 4.0064 (3.950)[4] |
| | Hf | 3.6153 (3.635)[4] | 3.7180 (3.748)[4] | 3.9606 (3.949)[5] |
| $d^1$ | V | 3.2668 (3.29)[6] | 3.3260(3.352)[4] | 3.6022 (3.595)[4] |
| | Nb | 3.3870 | 3.4845 | 3.6738 (3.642)[7] |
| | Ta | 3.3524 (3.346)[4] | 3.4602 (3.477)[4] | 3.6702 (3.651)[7] |
| $d^2$ | Mo | 3.1998 (3.201)[1] | 3.2685 (3.270)[1] | 3.4970 (3.469)[2] |
| | W | 3.1908 (3.222)[1] | 3.2574 | 3.4970 (3.496)[3] |
| $d^3$ | Tc | 3.0692 | 3.1543 | 3.4149 |
| | Re | 3.0750 | 3.1311 | 3.3834 |
| $d^5$ | Co | Non-Layered Structure | | 3.5983 (3.802)[8] |
| | Rh | | | 3.7563 (3.92)[8] |
| | Ir | | | 3.8431 (3.928)[8] |
| $d^6$ | Ni | 3.3174 | 3.4712 | 3.7248 (3.854)[8] |
| | Pd | 3.5408 | 3.6759 | 3.9162 (4.037)[8] |
| | Pt | 3.5237 (3.543)[4] | 3.6662 (3.728)[4] | 3.9554 (4.026)[8] |

## Supplementary references


1 Gordon, R., Yang, D., Crozier, E., Jiang, D. & Frindt, R. Structures of exfoliated single layers of WS 2, MoS 2, and MoSe 2 in aqueous suspension. *Physical Review B* **65**, 125407 (2002).

2 Dawson, W. & Bullett, D. Electronic structure and crystallography of MoTe2 and WTe2. *Journal of Physics C: Solid State Physics* **20**, 6159 (1987).

3 Brown, B. E. The crystal structures of WTe2 and high-temperature MoTe2. *Acta Crystallographica* **20**, 268-274 (1966).

4 Wilson, J. & Yoffe, A. The transition metal dichalcogenides discussion and interpretation of the observed optical, electrical and structural properties. *Advances in Physics* **18**, 193-335 (1969).

5 Smeggil, J. & Bartram, S. The preparation and x ray characterization of HfTe2− x, x= 0.061. *Journal of Solid State Chemistry* **5**, 391-394 (1972).

6 Gamble, F. Ionicity, atomic radii, and structure in the layered dichalcogenides of group IVb, Vb, and VIb transition metals. *Journal of Solid State Chemistry* **9**, 358-367 (1974).

7 Brown, B. E. The crystal structures of NbT2 and TaTe2. *Acta Crystallographica* **20**, 264-267 (1966).

8 Jobic, S., Brec, R. & Rouxel, J. Anionic polymeric bonds in transition metal ditellurides. *Journal of Solid State Chemistry* **96**, 169-180 (1992).
